# Past Climate Change and Plant Evolution in Western North America: A Case Study in Rosaceae

**DOI:** 10.1371/journal.pone.0050358

**Published:** 2012-12-07

**Authors:** Mats Töpel, Alexandre Antonelli, Chris Yesson, Bente Eriksen

**Affiliations:** 1 Department of Biological and Environmental Sciences, University of Gothenburg, Gothenburg, Sweden; 2 Gothenburg Botanical Garden, Gothenburg, Sweden; 3 Institute of Zoology, Zoological Society of London, London, United Kingdom; Institut de Biologia Evolutiva - Universitat Pompeu Fabra, Spain

## Abstract

Species in the ivesioid clade of *Potentilla* (Rosaceae) are endemic to western North America, an area that underwent widespread aridification during the global temperature decrease following the Mid-Miocene Climatic Optimum. Several morphological features interpreted as adaptations to drought are found in the clade, and many species occupy extremely dry habitats. Recent phylogenetic analyses have shown that the sister group of this clade is *Potentilla* section *Rivales*, a group with distinct moist habitat preferences. This has led to the hypothesis that the ivesioids (genera *Ivesia*, *Horkelia* and *Horkeliella*) diversified in response to the late Tertiary aridification of western North America. We used phyloclimatic modeling and a fossil-calibrated dated phylogeny of the family Rosaceae to investigate the evolution of the ivesioid clade. We have combined occurrence- and climate data from extant species, and used ancestral state reconstruction to model past climate preferences. These models have been projected into paleo-climatic scenarios in order to identify areas where the ivesioids may have occurred. Our analysis suggests a split between the ivesioids and *Potentilla* sect. *Rivales* around Late Oligocene/Early Miocene (∼23 million years ago, Ma), and that the ivesioids then diversified at a time when summer drought started to appear in the region. The clade is inferred to have originated on the western slopes of the Rocky Mountains from where a westward range expansion to the Sierra Nevada and the coast of California took place between ∼12-2 Ma. Our results support the idea that climatic changes in southwestern North America have played an important role in the evolution of the local flora, by means of *in situ* adaptation followed by diversification.

## Introduction

Understanding the influence of climate change on the evolution and distribution of the world's biota constitutes a major task in biology. An accurate estimation of how species have responded to changes in the past may enable us to better predict future responses to global warming, with far-reaching implications influencing the work of policy-makers and conservational biologists [1].

A suitable area for assessing the effect of climate change on plant evolution is western North America. This is a botanically diverse region, rich in both total species numbers and proportion of endemic species, and has undergone major climatic and geologic changes during the Cenozoic (the last 65 Ma). At the beginning of the Eocene (∼55.8-33.9 Ma) a warm and humid tropical climate prevailed in the region, but global cooling has since then gradually changed the conditions [2]. Onset of glaciation in Antarctica by the end of the Eocene was accompanied by rapid decline of global deep-sea temperatures [3]. Increased upwelling of cool Pacific ocean water off the Californian coast eventually led to summer drought by mid-Miocene (∼15 Ma) [4]. Global cooling also strengthened the westerlies [5], which increased winter precipitation after mid-Miocene (∼11.6 Ma). A Mediterranean type of climate, with summer droughts and winter precipitation, was in place in Late Miocene (∼10 Ma) [2]. Climate change in the area has been suggested to trigger the evolution of evening primroses (genus *Oenothera*, family Onagraceae) [6]–[7], but several questions remain concerning how general niche conservatism/lability has been in the area, and from which areas and habitat zones the local flora originated.

**Figure 1:**
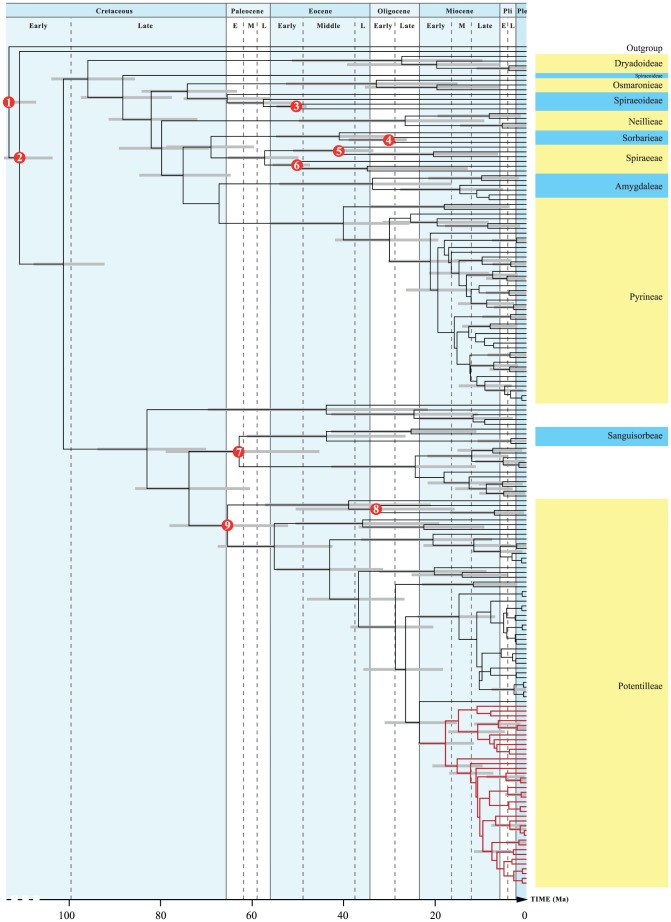
Molecular chronogram of Rosaceae. Maximum clade credibility tree obtained from 25000 post burn-in Bayesian chronograms generated in BEAST, with median branch lengths. Grey bars at nodes represent 95% Highest Posterior Densities of node ages. The red dots indicates age constraints used for the analysis; (1) The split between Rosales and Fabales was constrained to an age of 104–115 Ma based on a previous analysis [31], and (2) a *Crataegites borealis* fossil was used to set a conservative minimum age of 85.8 Ma on Rosaceae [32], [34]. Subclades of Rosaceae were calibrated using fossil data from (3) *Neviusia*, 48.7 Ma [35], (4) *Chamaebatiaria*, 26.85 Ma [40], (5) *Holodiscus*, 34.1 Ma [37], (6) *Spiraea*, 48 Ma [35], (7) *Rosa*, 34.1 Ma [38], (8) *Fragaria*, 2.5 Ma [45], (9) *Potentilla* 11.6 Ma [42]. A uniform prior with a maximum age of 115 Ma was used for all calibration points. Also indicated are the tribes of Rosaceae (species highlighted in blue and yellow) as well as the ivesioid clade highlighted in red. Time scale from [61].

The ‘ivesioids’ are a well-supported plant clade [8]–[9] confined to western North America [10]. It is nested within *Potentilla* L. (cinquefoil) in the Rosaceae – a cosmopolitan family of large ecological and economic importance, which includes many edible fruits (apples, plums, cherries, pears, strawberries, almonds) as well as ornamentals (roses, firethorns, hawthorns). As currently circumscribed (Figures 1 and S1; [8]–[9], [11]–[12]), the ivesioid clade includes more than 50 species classified in three genera: *Ivesia*, *Horkelia* and *Horkeliella* [10], [13]–[14]. Common to many of them is that they grow under extremely dry conditions and have developed means to avoid drought (petrophily on protected rock faces, tolerance of alkalinity) or minimize water loss (increased pubescence, numerous minute leaflet segments in a tightly overlapping arrangement).

**Figure 2:**
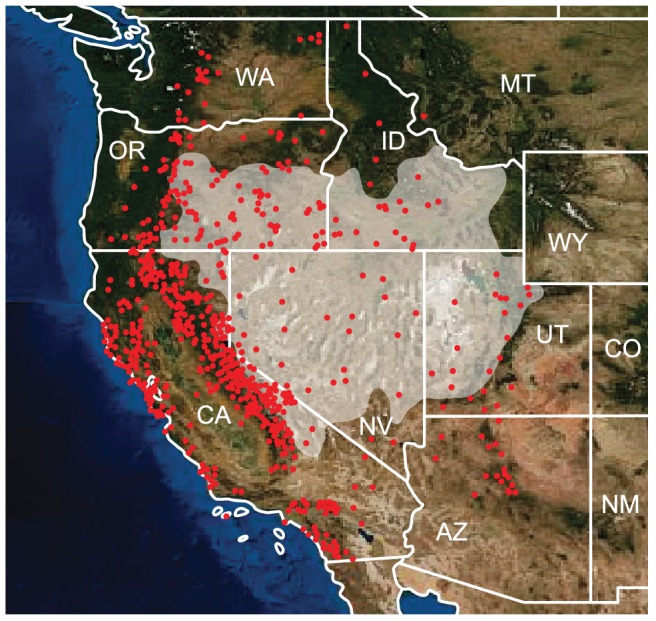
Geographical distribution of the ivesioids. Shaded area represents the exten of the Great Basin. Locations where climate data were sampled for the bioclimatic models for extant species are marked with dots. State names are abbreviated: AZ – Arizona, CA – California, CO – Colorado, ID – Idaho, MT – Montana, NE – Nevada, NM – New Mexico, OR – Oregon, UT – Utah, WA – Washington and WY – Wyoming. Background image by www.earthobservatory.nasa.gov.

*Potentilla* sect. *Rivales* is the sister group of the ivesioids [8]–[9]. Species in this group preferably occupy seasonally inundated flats or lake and stream shores, and have a widespread distribution in the Northern hemisphere. In contrast, the ivesioid species usually reside in extremely arid regions, alpine habitats and sites with a Mediterranean type of climate in the Great Basin (Figure 2) and adjacent arid parts of western North America, and comprise many narrowly endemic species [10], [13]–[14].

Phyloclimatic modeling [7], [15]–[19] combines phylogenetic estimation of species relationships with bioclimatic models [20]. These models use climate data from known species locations to predict areas of suitable climate for that species, by projecting the models into a present-day climatic scenario. They can thus estimate the total potential distribution of species even when not all localities and populations have been sampled. Furthermore, different methods for ancestral state reconstruction can be used to reconstruct the climatic preferences for ancestral nodes in a dated phylogeny. Historical distributions regulated by climatic conditions can then be estimated by projecting the optimized models into past climate scenarios, leading to an estimate of ancestral distributions. These models can thus be used to evaluate the evolutionary importance of niche conservatism for producing the distribution of plant diversity seen today (e.g., [21]), and help predict how this diversity may be affected in the future by global warming.

The primary objective of this study is to test the hypothesis that species in the ivesioid clade evolved in response to late Tertiary development of dry conditions in western North America. Under such circumstances, we would expect its stem node to have originated in western North America, and that the crown age of this clade - reflecting the onset of diversification of dry-adapted species - is not older than the proposed time of the aridification in the region. To address this, we have performed a molecular dating analysis of a plastid phylogeny of Rosaceae to establish the age of the ivesioid clade and produced niche models for both extant species and well-supported nodes of the phylogeny. Projections of these models into palaeoclimatic scenarios were used to estimate the geographic origin of the group and to infer changes in geographical distributions over time.

## Materials and Methods

### Molecular data

A taxonomically representative set of sequences (selected to represent all subfamilies of Rosaceae; see Table 1) from the plastid matK and trnL-trnF intergenic spacer was downloaded from the National Center for Biotechnology Information (www.ncbi.nlm.nih.gov). *Pisum sativum* (Fabaceae) was chosen as outgroup for the analysis, and *Rhamnus cathartica* (Rhamanaceae) was also included as representative for another family in the order Rosales.

**Table 1:** NCBI accession numbers for the sequences used in the phylogenetic inference and dating analysis.

Species	matK	trnL/F
Adenostoma fasciculatum	AF288093	AF348535
Agrimonia nipponica	AB073682	-
Agrimonia pilosa	AB012001	-
Alchemilla japonica	AB073681	EU072631
Amelanchier bartramiana	DQ860450	DQ863222
Amelanchier utahensis	EU025916	-
Aria alnifolia	DQ860451	DQ863223
Aruncus dioicus	AF288094	AF348536
Cercocarpus betuloides	AF288095	AF348537
Cercocarpus montanus	EU025929	-
Chaenomeles cathayensis	DQ860453	DQ863225
Chaenomeles sinensis	DQ860471	DQ863243
Chamaebatia foliolosa	AF288096	AF348538
Chamaebatiaria millefolium	AF288097	AF348539
Chamaemeles coriacea	DQ860454	DQ863226
Chamaemespilus alpina	DQ860455	DQ863227
Coleogyne ramosissima	DQ851224	-
Comarella multifoliolata	-	FN556394
Cormus domestica	DQ860456	DQ863228
Cotoneaster coriaceus	DQ860457	DQ863229
Cotoneaster pannosus	AF288098	AF348540
Crataegus monogyna	AF288099	-
Crataegus submollis	DQ860458	-
Cydonia oblonga	DQ860459	DQ863231
Dichotomanthes tristaniicarpa	DQ860460	DQ863232
Docyniopsis tschonoskii	DQ860461	DQ863233
Drymocallis glandulosa	EU025919	-
Eriobotrya japonica	DQ860462	DQ863234
Exochorda racemosa	AF288100	AF348542
Fragaria iinumae	AB073685	AF348544
Fragaria vesca	AF288102	AF348545
Gillenia stipulata	AF288103	AF348554
Gillenia trifoliata	AF288104	AF348555
Heteromeles arbutifolia	DQ860464	DQ863236
Holodiscus discolor	AF288105	AF348546
Horkelia bolanderi	-	FN556395
Horkelia californica	-	FR872958
Horkelia cuneata	-	FR872977
Horkelia daucifolia	-	FR872995
Horkelia fusca	-	FR872964
Horkelia hendersonii	-	FR872940
Horkelia hispidula	-	FR872985
Horkelia marinensis	-	FR872972
Horkelia rydbergii	-	FR872981
Horkelia tenuiloba	-	FR872942
Horkelia tridentata	FR851329	FR872969
Horkelia truncata	-	FR872978
Horkelia wilderae	-	FR872980
Horkelia yadonii	-	FR872941
Horkeliella congdonis	-	FR872976
Horkeliella purpurascens	-	FR872976
Ivesia aperta	-	FR872987
Ivesia argyrocoma	-	FR873003
Ivesia arizonica arizonica	FR851326	FR872960
Ivesia bailey beneolens	FR851327	FR872954
Ivesia cryptocaulis	-	FR872968
Ivesia jaegeri	-	FR872983
Ivesia kingii kingii	FR851328	FR872965
Ivesia longibracteata	-	FR872937
Ivesia lycopodioides	FR851324	FR872967
Ivesia pygmea	FR851335	FR872963
Ivesia rhypara	FR851332	FR872953
Ivesia sabulosa	FR851334	FR872956
Ivesia santolinoides	-	FR872984
Ivesia saxosa	FR851336	FR872966
Ivesia sericoleuca	-	FR872989
Ivesia setosa	-	FR872957
Ivesia shockleyi	FR851333	FR872955
Ivesia tweedyi	-	FR872944
Ivesia unguiculata	FR851331	FR872952
Ivesia utahensis	-	FR872946
Ivesia webberi	-	FR872988
Kageneckia angustifolia	DQ860447	DQ863219
Kageneckia oblonga	AF288106	AF348547
Kelseya uniflora	DQ851226	DQ851232
Kerria japonica	AB073686	-
Lindleya mespiloides	DQ860448	DQ863220
Luetkea pectinata	DQ851227	DQ851233
Lyonothamnus floribundus	AF288107	AF348548
Maddenia hypoleuca	DQ851228	AY864827
Malacomeles denticulata	DQ860465	DQ863237
Malus sargentii	DQ860466	DQ863238
Malus sylvestris	AM042563	-
Malus trilobata	DQ860463	DQ863235
Malus X domestica	AM042561	-
Mespilus germanica	DQ860467	DQ863239
Neillia thyrsiflora	AF288108	AF348549
Neviusia alabamensis	AF288109	AF348550
Oemleria cerasiformis	AF288110	AF348551
Osteomeles schwerinae	DQ860468	DQ863240
Peraphyllum ramosissimum	DQ860469	DQ863241
Petrophyton caespitosum	DQ851229	DQ851234
Photinia pyrifolia	DQ860452	DQ863224
Photinia serrulata	AF288111	-
Photinia villosa	DQ860470	DQ863242
Physocarpus alternans	EU025931	AY555407
Physocarpus capitatus	AF288112	AF348553
Pisum sativum	AY386961	AY839473
Potentilla alba	-	FN556397
Potentilla anserina	AF288113	FN561752
Potentilla apennina	-	FR872950
Potentilla arizonica	FR851325	FR872974
Potentilla atrosanguinea	-	FN561744
Potentilla biennis	EU025921	FR872938
Potentilla caulescens	-	FN561737
Potentilla clusiana	-	FN556401
Potentilla consinna	EU025923	-
Potentilla crassinervia	FR851330	FR872951
Potentilla diversifolia	EU025930	-
Potentilla elegans	-	FN556404
Potentilla erecta	-	FN556405
Potentilla flabellifolia	-	FN556406
Potentilla fragarioides	AB073687	FN561747
Potentilla fragiformis	-	FN556407
Potentilla gorodkovii	-	FN556408
Potentilla gracilis	EU025933	FN556464
Potentilla grandiflora	-	FN556465
Potentilla hyparctica	-	FN556410
Potentilla indica	-	AJ512242
Potentilla lignosa	-	FJ422299
Potentilla microphylla	-	FN556412
Potentilla morefeildii	-	FR872962
Potentilla newberryi	-	FR872993
Potentilla norvegica	-	FN561730
Potentilla pedersenii	-	FN556415
Potentilla pensylvanica	EU025932	FN556416
Potentilla pulvinaris	-	FN556418
Potentilla reptans	FJ395425	AJ512241
Potentilla rivalis	-	FR872992
Potentilla saxifraga	-	FR872948
Potentilla speciosa	-	FR872943
Potentilla stolonifera	-	FN556420
Potentilla subvahliana	-	FN556421
Potentilla supina	-	FR872945
Potentilla thuringiaca	-	FN556423
Potentilla uniflora	-	FN556425
Potentilla valderia	-	FR872949
Prinsepia sinensis	AF288114	AF348558
Prunus dulcis	AF288115	-
Prunus laurocerasus	AF288116	AF348559
Prunus persica	AF288117	AF348560
Prunus virginiana	AF288118	-
Purshia tridentata	AF288119	AF348562
Pygeum topengii	DQ851230	-
Pyracantha coccinea	DQ860472	DQ863244
Pyrus caucasica	AF288120	-
Pyrus communis	DQ860473	-
Rhamnus cathartica	AY257533	-
Rhaphiolepis indica	DQ860474	DQ863246
Rhodotypos scandens	AF288122	AF348566
Rosa acicularis	AB039293	DQ778872
Rosa brunonii	AB039312	-
Rosa cymosa	AB039317	DQ778846
Rosa foetida	AB011975	DQ778850
Rosa gallica	AB011978	DQ778852
Rosa marretii	AB039297	DQ778863
Rosa multiflora	AB011991	DQ778870
Rosa platyacantha	AB039291	-
Rosa spinosissima	AB011976	DQ778886
Rosa stellata	AB039322	-
Rosa willmottiae	AB039298	DQ778892
Rubus arizonensis	EU025927	-
Rubus chamaemorus	AY366358	AJ416464
Rubus discolor	EU025922	-
Rubus trifidus	AB039323	-
Rubus ursinus	AF288124	AF348568
Sanguisorba annua	AB073689	AY634767
Sanguisorba filiformis	AB073690	AY634769
Sorbaria sorbifolia	AF288125	AF348569
Sorbus americana	DQ860475	DQ863247
Sorbus californica	AF288126	AF348570
Spiraea cantoniensis	AF288127	-
Stephanandra chinensis	AF288128	AF348572
Stranvaesia davidiana	DQ860476	DQ863248
Torminalis clusii	DQ860477	DQ863249
Vauquelinia californica	AF288129	DQ863221

In addition, new sequences from species in genus *Potentilla*, including the ivesioid clade, were generated. The *matK* region was amplified and sequenced with the trnk-3914 FM primer [22] and the matK2R primer [23]. The *trnL* intron and the *trnL-trnF* intergenic spacer were amplified with the trnLc and trnLf primers [24]. Two additional primers, trnLe and trnLd, together with the PCR amplification primers were used for sequencing. The PCR amplification of the two regions was performed using 12.5 µl MasterAmp 2× PCR PreMix G (Epicentre Biotechnologies, Madison, Wisconsin, USA), 0.6 µM of the forward and reverse primers, 1 unit Thermoprime Plus DNA Polymerase (ABgene House, Epsom, UK), 1 µl template DNA and purified water to a final volume of 25 µl.

The PCR mix was heated to 95°C for 5 minutes followed by 35–45 cycles of a denaturation step at 95°C for 30 seconds, annealing at 55°C for 30 seconds and extension for 1 minute (*trnL/F*) or 2 minutes (*matK*) at 72°C. The program ended with an additional 10 minutes (*trnL/F*) or 7 minutes (*matK*) extension step at 72°C. The resulting PCR products were sequenced by Macrogen Inc. (Seoul, Korea). The *matK* and *trnL/F* sequences (Table 1) were then aligned separately with mafft-linsi v.6.717b [25] and subsequently concatenated into a common matrix.

### Phylogenetic inference and Molecular dating

We investigated whether the sequences evolved in a clocklike way by generating a neighbor joining tree in PAUP [26] and comparing Maximum Likelihood scores calculated from the data with and without enforcing a molecular clock. A likelihood ratio (LR) test was then performed with LR = 2 (L_mol. clock enforced_−L_no mol. clock enforced_) and assumed to be distributed as a χ^2^ with S-2 degrees of freedom, S being the number of taxa in the dataset. Since the LR test rejected a molecular clock (*p*<0.001), we chose to estimate divergence times with the relaxed clock algorithm implemented in the software BEAST v.1.6.1 [27] using the beagle library for likelihood calculations [28]. Fourteen runs of 10 million generations were performed, assuming an uncorrelated lognormal clock model and a pure birth (Yule) process under the GTR+Γ model, sampling every 2500^th^ generation. The nucleotide substitution model was selected using the program MrAic [29] and the Aikaike information criterion. Performance of the analysis (convergence of the independent runs and effective sample sizes for all sampled parameters) was evaluated using Tracer v.1.5 [30], after which 2500 trees were removed from each of the fourteen tree sets as the initial burn-in. Median and 95% Highest Posterior Density (HPD) intervals of node ages were then calculated from the remaining 21000 trees using the software TreeAnnotator v.1.6.1 [28].

#### Calibration

The crown age of the tree, corresponding to the split between Fabales (here represented by *Pisum*) and Rosales (all other species), was set as a uniform prior between 104 and 115 Ma. This interval corresponds to the lower age estimate for the Rosales stem lineage and the upper age estimate for the Rosid crown group, respectively, as inferred in BEAST in a large fossil-based dating analysis of the Rosids [31]. Although this maximum age may be incorrect, in the absence of further evidence we consider this a conservative assumption since the Rosales clade has a well-supported position within the Rosids (Figure 1 in [31]). In addition, seven carefully chosen fossils were used to impose minimal age constraints on the prior distributions.

The oldest fossil assigned to the crown group of Rosaceae is *Crataegites borealis* [32] from the Kolyma area in Siberia. It belongs to the Bour-kemuss Formation of the Zyrianka Coal Basin and has been dated to Early Albian (99.6–112 Ma) in the Cretaceous, by stratigraphic methods ([33] and references therein). The ^40^Ar/^39^Ar dates for the geographically adjacent but stratigraphically younger Chauna group tephra was determined to fall within the Coniacian stage (85.8–89.3 Ma) in late Cretaceous [34]. *Crataegites borealis* is based on a number of very well preserved leaf imprints [32]. The similarity to modern-day leaves of *Crataegus* is striking and there is no obvious reason to dispute the taxonomic position of the fossils in the crown group of Rosaceae.

Fossils of *Spiraea* (Amygdaloideae) and *Neviusia* (Rosoideae) were found at Republic, Washington, USA [35] and dated to 48–49 Ma [36]. Representatives of the genera *Holodiscus* (Amygdaloideae; [37]) and *Rosa* (Rosoideae; [38]) are known from Florissant, Colorado and are dated to 34.1 Ma in Late Eocene [39]. *Chamaebatiaria* (Rosoideae) fossils belong to the Creede Flora, Colorado [40], and the formation in which they were found has been dated to early Late Oligocene (26.85 Ma [41]). The oldest fossils of the genus *Potentilla* (Rosoideae; [42]) are from brown coal strata in Lausitz, Germany, formed in Early–Middle Miocene (11.6–23.0 Ma; [43]). Reference to an older *Potentilla* fossil is given by Wolfe and Schorn [44] from the Creede Flora in North America (27.3 Ma). The fossil, a leaf imprint, was originally described as a member of Ranunculaceae by Axelrod [40] but was reclassified to *Potentilla*/Rosaceae by Wolfe and Schorn [44]. We have examined the photography of this fossil and dispute its reclassification, choosing instead the younger European fossil for calibration of the genus. Macrofossils of *Fragaria* (Rosoideae) were found in the Beaufort formation, Prince Patrick Island in the Canadian Arctic [45]. The Beaufort formation is considered to be of the same age as the Lost Chicken tephra in Alaska dated to 2.9±0.4 Ma [46].

### Species distribution data

Locality data for ivesioid species were downloaded from the Global Biodiversity Information Facility portal (www.gbif.org), Jepson Online interchange (ucjeps.berkeley.edu/interchange.html) and the Consortium of Pacific Northwest Herbaria websites (www.pnwherbaria.org). Duplicated data points were removed manually. If, in total, less than ten locations were found in the online databases, more locality data were collected from herbarium labels. The occurrence data was then plotted in a GIS using QGIS (http://qgis.org/), to verify that it agreed with current known distributions. Data points were this way cleaned by visual inspection (e.g. samples from coastal species ending up in the ocean were excluded).

### Climate scenarios

Climate datasets for present day conditions (experiment set named *xakxu*) and paleoclimatic scenarios for 10 Ma (*xakfl*), 8 Ma (*xakxu*) and 3 Ma (*xaiud*) were provided by the BRIDGE project (www.bridge.bris.ac.uk/resources/simulations). Each dataset contained the sixteen climate variables listed in Table 2.

**Table 2:** The sixteen climate variables that where considered for the analysis, listed with mean AUC values. Variables used in the final analysis are indicated with *.

	Climate variables	Mean AUC	1	2	3	4	5	6	7	8	9	10	11	12	13	14	15
**1**	Mean_temperature	0,96															
**2**	Mean_temperature_in_warmest_month	0,90	0,61														
**3**	Mean_temperature_in_coolest_month*	0,97	0,87	0,20													
**4**	Standard_deviation_of_mean_temperature*	0,88	−0,05	0,73	−0,46												
**5**	Mean_daily_precipitation	0,80	−0,34	−0,27	−0,14	−0,07											
**6**	Mean_daily_precipitation_in_wettest_month	0,80	−0,22	−0,24	−0,01	−0,12	0,97										
**7**	Mean_daily_precipitation_in_warmest_month*	0,87	−0,21	0,21	−0,44	0,55	0,10	−0,05									
**8**	Mean_daily_precipitation_in_driest_month	0,81	−0,32	0,12	−0,51	0,53	0,19	0,01	0,97								
**9**	Mean_daily_precipitation_in_coolest_month*	0,90	−0,15	−0,21	0,07	−0,15	0,95	0,99	−0,10	−0,04							
**10**	Standard_deviation_of_mean_precipitation	0,83	−0,19	−0,19	0,00	−0,08	0,94	0,99	−0,09	−0,03	0,99						
**11**	Mean_daily_precipitation_in_coolest_quarter	0,87	−0,20	−0,22	0,01	−0,12	0,97	0,99	−0,06	0,01	1,00	0,99					
**12**	Mean_daily_precipitation_in_driest_quarter	0,80	−0,34	−0,03	−0,43	0,33	0,34	0,15	0,88	0,94	0,11	0,10	0,16				
**13**	Mean_daily_precipitation_in_warmest_quarter	0,86	−0,34	−0,06	−0,43	0,30	0,29	0,09	0,87	0,93	0,06	0,04	0,10	0,99			
**14**	Mean_daily_precipitation_in_wettest_quarter	0,80	−0,25	−0,25	−0,05	−0,11	0,98	1,00	−0,02	0,06	0,98	0,99	0,99	0,20	0,14		
**15**	Mean_temperature_in_coolest_quarter	0,97	0,91	0,26	0,99	−0,40	−0,21	−0,09	−0,39	−0,47	−0,01	−0,08	−0,06	−0,41	−0,40	−0,12	
**16**	Mean_temperature_in_warmest_quarter	0,91	0,71	0,99	0,32	0,64	−0,31	−0,26	0,18	0,08	−0,22	−0,21	−0,24	−0,05	−0,08	−0,27	0,38

### Selection of climate variables

Various strategies have been proposed for selecting climate variables to use in bioclimatic modeling. Methods for selecting or rejecting variables have included the quantification of variable contribution to the model, or specifically for phyloclimatic modeling [19], an assessment of the phylogenetic conservatism of individual variables, but most have investigated the correlation of prospective variables [15], [47]–[48]. Correlated climate variables will emphasize certain climate components (e.g. temperature or precipitation) if included in the analysis, and potentially result in incorrect inference of climate models. Thuiller [48] used principal component analysis to select uncorrelated variables for the models and Beaumont et al. [47], evaluated several different methods, including random sampling of variables, to assess the extent to which parameter choice influenced the predicted areas. The latter investigation showed that the size of the predicted area of distribution decreased when more climate variables were included in the analysis. Hence, selecting climate variables is an important step in inference of ancestral distribution areas.

We have used a novel method to exclude correlated variables while taking their prediction power in the form of Area under the Receiver Operating Characteristic (AUC) values into account. AUC is a measure of how well a model discriminates between sites where a species is present, compared to where it is absent [49]. The values range from 0 to 1, where a score of 1 indicates a perfect prediction of distribution and a score of 0.5 equals a random prediction of sites [50]. We produced bioclimatic models for each of the 38 species in the ivesioid clade, using one of the sixteen variables at a time. We recorded the AUC value for each model, and hence, each climate variable given a particular species, and the number of environmentally unique occurrence points for each analysis. An environmentally unique occurrence point is a species location with a value not previously sampled by the projected model. We then calculated the mean AUC value from each analysis with ten or more environmentally unique locations.

Correlation between the sixteen climate variables was then assessed using the function *cor* in the statistical package R [51]. Variable pairs with correlation coefficients greater than 0,8 were identified and the climate variable with the lowest AUC value was excluded (Table 2). The four variables remaining after this exclusion process were used to build the bioclimatic models for the extant species and the ancestral nodes.

### Bioclimatic models for extant species

Locality data together with the four selected climate variables (Table 2) were used to define the climate preferences for each of the 38 ivesioid species, using the Envelope Score algorithm implemented in OpenModeller v.1.1.0 (openmodeller.sourceforge.net). The Envelope score is a modified version of the Bioclimatic Envelope Algorithm (Bioclim) that uses the observed maximum and minimum values in each environmental variable to determine the climate preferences for a taxon [20]. These preferences, called the bioclimatic envelope, can then be projected into a climate scenario to identify areas with a suitable climate for the taxon. The probability of a suitable environment in the projected model is determined by the number of layers with a value within the min-max threshold, divided by the total number of layers in the model [52].

The Bioclim methodology treats the environmental parameters independently of each other. This is a prerequisite for the ancestral state reconstruction where each variable in the bioclimatic envelop has to be optimized independently with currently available methods. Also, the simplicity of the algorithm makes it possible to combine these optimized variables to an ancestral bioclimatic envelope. More complex algorithms do not permit this independent treatment of variables as they attempt to account for the correlation between variables, and have therefore not been used for phylogenetic niche modeling [19].

### Ancestral state reconstruction

**Figure 3:**
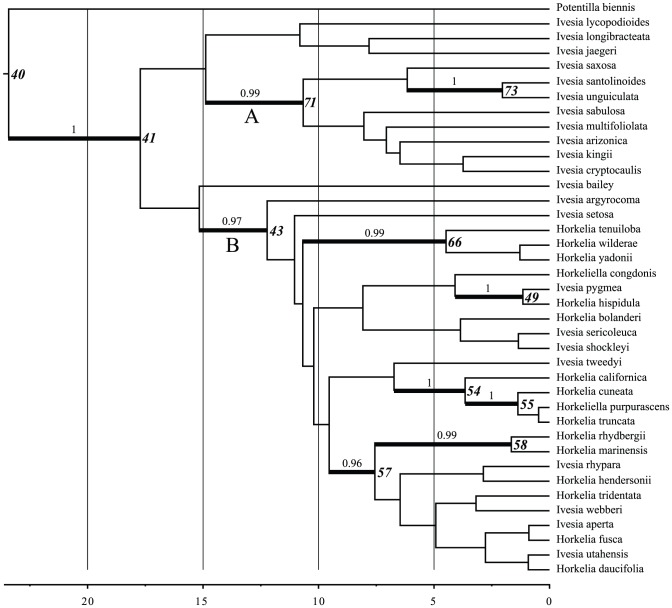
The ivesioid clade with node numbers indicated. Posterior probability (pp.) greater than 0.5 is shown. Branches with pp. greater than 0.95 are shown with thicker lines. Nodes for which ancestral models were projected into climate scenarios are indicated with numbers in boldface.

Ancestral climate preferences were reconstructed for each node in the ivesioid phylogeny (Figures 3 and S2) using the function *ace* in the package *ape* [53] of the statistical program R [51]. Independent optimizations were done for the maximum and the minimum values of each variable by fitting a Brownian motion model using Maximum Likelihood optimization [54]. Optimized models for each node are presented in table 3.

**Table 3:** Optimized ancestral maximum and minimum values for the four climate variables used. Temperatures are given in °C and precipitation in millimeters.

	Standard deviation of mean temperature		Mean daily precipitation in warmest month		Mean daily precipitation in coolest month		Mean temperature in coolest month	
None nr.	Max	Min	Max	Min	Max	Min	Max	Min
40	10,3	7,1	1,3	0,1	5,5	0,1	3,6	−5,0
41	9,8	7,4	0,6	0,1	4,8	0,1	2,8	−3,3
42	9,7	7,2	0,5	0,1	4,8	0,1	2,5	−2,9
43	9,4	6,9	0,4	0,1	4,7	0,1	3,0	−1,7
44	9,3	6,6	0,4	0,1	5,0	0,1	3,2	−1,5
45	9,2	6,4	0,4	0,1	5,1	0,1	3,4	−1,3
46	9,1	6,3	0,4	0,1	5,2	0,1	3,4	−1,3
47	8,9	6,3	0,3	0,1	5,2	0,1	3,1	−1,2
48	8,5	6,9	0,2	0,1	4,7	0,1	2,0	−1,1
49	8,9	7,1	0,1	0,1	4,6	0,1	1,4	−1,7
50	8,9	5,8	0,3	0,1	5,9	0,1	3,5	−0,9
51	9,6	7,6	0,3	0,1	5,3	0,1	0,0	−4,1
52	9,1	6,2	0,4	0,1	5,3	0,1	3,6	−1,4
53	9,0	5,5	0,5	0,1	5,4	0,1	4,6	−1,7
54	9,1	4,3	0,4	0,1	5,2	0,1	7,5	0,4
55	9,0	4,6	0,4	0,0	3,6	0,0	8,5	2,3
56	8,7	5,9	0,2	0,0	2,7	0,0	7,2	2,4
57	9,0	6,3	0,4	0,1	5,8	0,1	3,5	−1,4
58	8,8	5,7	0,3	0,0	4,6	0,0	6,6	2,7
59	8,9	6,4	0,4	0,1	6,3	0,1	2,8	−2,2
60	8,5	6,0	0,3	0,1	7,8	0,1	2,8	−2,3
61	9,1	6,8	0,3	0,1	6,3	0,1	1,9	−3,2
62	9,0	6,8	0,3	0,1	6,5	0,1	2,0	−3,0
63	9,3	7,5	0,4	0,1	6,0	0,1	0,5	−4,9
64	9,5	7,4	0,5	0,1	6,5	0,1	0,8	−5,5
65	9,4	8,1	0,4	0,2	5,3	0,2	−1,1	−5,8
66	8,7	5,0	0,4	0,1	4,7	0,1	6,8	2,5
67	9,2	6,1	0,3	0,1	2,7	0,1	6,7	3,3
68	9,7	7,7	0,5	0,1	4,4	0,1	2,7	−2,9
69	9,4	7,7	0,3	0,1	4,6	0,1	3,3	−2,1
70	9,2	7,8	0,3	0,1	4,7	0,1	3,3	−1,4
71	10,0	8,1	0,4	0,1	3,7	0,1	2,2	−3,1
72	9,8	7,5	0,2	0,1	4,2	0,1	3,8	−1,9
73	9,5	7,2	0,1	0,1	4,6	0,1	3,3	−1,5
74	10,2	8,7	0,4	0,1	3,0	0,1	0,8	−3,9
75	10,2	8,9	0,4	0,2	2,8	0,2	0,5	−3,8
76	10,3	8,8	0,4	0,2	2,8	0,2	0,3	−4,1
77	10,2	8,6	0,3	0,1	3,0	0,1	−0,3	−4,5

Node numbers corresponds to the numbers indicated in figure S2.

### Ancestral bioclimatic model

The optimized maximum and minimum values for the four climate variables were used to build bioclimatic models for all nodes in the ivesioid clade. Models for nodes with a posterior probability higher than 0.95 were then projected in to the climate scenario that corresponded best in time with the age of the nodes as follows: node 40, 41, 43 and 71 were projected in to the climate scenario for 10 Ma; node 57 in a 8 Ma scenario; node 49, 54, 55, 58, 66 and 73 in the climate scenario for 3 Ma.

The same models were also projected into present-day climate data to evaluate whether the variation in predicted geographic area between nodes in the tree depends on variation in the climate scenarios or in the inferred models. By keeping one of these variables constant (in this case the climate scenario), any variation in the inferred area with a suitable climate will depend on the inferred model. Differences between optimised models can that way be visualised. This analysis was performed to identify shifts in climate preferences during the evolution of the ivesioids. Additionally, the comparison of models for extant taxa projected into the present-day climate scenario, with ancestral niches projected into present-day climate scenarios permits a visual comparison of the differences between the extant and ancestral niches.

### Test of models

AUC values for all niche models of extant taxa were calculated. A test of the correlation of the projected surfaces for all extant taxa was performed using the niche.overlap tool in the phyloclim package [55] of R [51]. Additionally, the age.range.correlation tool (also in phyloclim) was used to test for correlation between the niche overlap of two taxa and age to their most recent common ancestor (MRCA).

## Results

### Phylogenetic inference and Molecular dating

The dated phylogeny of the Rosaceae family (Figures 1 and S1) identified the three subfamilies Rosoideae, Amygdaloideae and Dryadoideae as monophyletic. Except for the position of the species *Lyonothamnus floribundus*, the tribes presented by Potter et al. [56] were also in congruence with our phylogeny. *Potentilla*, and the ivesioids were inferred to be monophyletic.

The estimated 95% Highest Posterior Density (HPD) of the crown age of Rosaceae was 108.3-92.9 Ma (median 101.3 Ma). The tribe Potentilleae split off from Sanguisorbeae and *Rosa* 86.2-61.2 Ma (median 73.8 Ma) and the split between *Potentilla* and Fragariinae happened 78.7-52.8 Ma (median 65.4 Ma). The *Potentilla* crown group diversified between 68.2-43.1 Ma (median 55.2 Ma). Furthermore, the ivesioids formed a well-supported clade (pp. 1) and had a stem age of 31.6-15.9 Ma (median 23.4 Ma). Support for the internal topology of the clade was low, with a few exceptions. Ten clades with a posterior probability greater than 0.95 were identified and selected for further investigations (figure 3). Our results show that the ivesioids diversified between 24.3-12.1 Ma (median 17.7 Ma) into a clade with a mainly eastern (present day) Great Basin distribution (clade A; Figure 3), and a clade more or less confined to the Sierra Nevada and California in the west (clade B; Figure 3).

### Bioclimatic models

#### Species distribution data

The number of occurrence points used varied between 10 and 256 for 36 of the 38 species (Table 4). Locality data for two species, *Ivesia longibracteata* (five points) and *I. cryptocaulis* (two points), were still less than the desired ten data points after the dataset had been complemented with data from herbarium collections.

**Table 4:** Number of occurrence points for the 38 ivesioids and the maximum and minimum values for the four climate variables in their models.

Species	Nr, of locations	Standard deviation of mean temperature		Mean daily precipitation in warmest month		Mean daily precipitation in coolest month		Mean temperature in coolest month	
		Max	Min	Max	Min	Max	Min	Max	Min
Horkelia bolanderi	14	8,0	2,5	0,4	0,0	7,3	0,5	9,4	4,2
Horkelia californica	123	9,4	2,5	0,5	0,0	7,3	1,3	9,4	−0,1
Horkelia cuneata	42	9,4	2,8	0,5	0,0	4,1	0,7	11,0	3,2
Horkelia daucifolia	35	8,2	5,8	0,3	0,0	8,4	5,3	4,2	−4,0
Horkelia fusca	256	10,0	5,8	1,0	0,0	8,4	1,5	4,6	−8,5
Horkelia hendersonii	11	7,2	5,8	0,3	0,1	8,4	4,2	2,9	0,8
Horkelia hispidula	11	8,9	7,2	0,1	0,1	4,2	2,2	0,8	−0,8
Horkelia marinensis	16	7,8	2,5	0,4	0,0	7,3	4,8	9,4	4,2
Horkeliella congdonis	15	7,6	7,2	0,1	0,1	4,4	3,5	1,8	−0,1
Horkeliella purpurascens	20	8,9	7,2	0,1	0,0	4,2	1,5	4,6	−0,8
Horkelia rydbergii	39	9,9	8,7	0,1	0,0	1,5	1,2	4,6	2,4
Horkelia tenuiloba	18	7,8	2,5	0,4	0,0	7,3	4,8	9,4	4,2
Horkelia tridentata	116	9,0	5,8	0,3	0,0	8,4	3,5	4,2	−2,8
Horkelia truncata	24	8,4	5,3	0,3	0,0	0,7	0,5	9,2	5,7
Horkelia wilderae	10	9,9	9,9	0,1	0,1	1,2	1,2	2,4	2,4
Horkelia yadonii	10	8,7	2,8	0,5	0,0	3,4	0,8	11,0	4,6
Ivesia aperta	26	9,0	9,0	0,1	0,1	4,9	4,9	−2,8	−2,8
Ivesia argyrocoma	25	9,9	9,4	0,1	0,1	1,3	1,2	3,2	2,4
Ivesia arizonica	11	10,5	8,7	0,5	0,1	2,5	1,0	0,1	−5,7
Ivesia baileyi	51	10,5	7,8	0,4	0,1	5,3	1,2	−2,1	−7,1
Ivesia cryptocaulis	2	9,8	9,8	0,2	0,2	1,4	1,4	−2,3	−2,3
Ivesia jaegeri	13	9,9	9,8	0,2	0,1	1,4	1,2	2,4	−2,3
Ivesia kingii	24	10,6	7,2	0,5	0,1	4,9	1,0	0,8	−7,1
Ivesia longibracteata	5	8,2	6,3	0,2	0,0	8,1	6,4	4,2	1,6
Ivesia lycopodioides	83	9,0	7,2	0,1	0,0	4,9	1,5	4,6	−2,8
Ivesia multifoliolata	12	10,3	10,3	0,3	0,3	1,0	1,0	−0,3	−0,3
Ivesia pygmaea	32	9,0	7,2	0,1	0,1	4,9	3,5	1,8	−2,8
Ivesia rhypara	29	9,4	5,8	0,4	0,1	8,4	1,8	2,7	−5,5
Ivesia sabulosa	19	10,6	9,7	0,5	0,2	2,4	1,0	−0,3	−7,1
Ivesia santolinoides	57	9,9	7,2	0,1	0,0	4,4	1,2	4,6	−0,1
Ivesia saxosa	20	9,9	7,2	0,1	0,0	4,2	0,5	6,8	−0,8
Ivesia sericoleuca	23	9,0	9,0	0,1	0,1	4,9	4,9	−2,8	−2,8
Ivesia setosa	41	10,5	7,8	0,4	0,1	5,3	1,2	−2,1	−7,1
Ivesia shockleyi	27	10,5	7,2	0,4	0,1	5,3	1,3	0,8	−7,1
Ivesia tweedyi	36	8,4	6,2	1,0	0,3	6,2	3,2	0,4	−6,9
Ivesia unguiculata	15	9,0	7,2	0,1	0,1	4,9	3,5	1,8	−2,8
Ivesia utahensis	10	10,6	10,6	0,5	0,4	1,8	1,0	−7,1	−8,2
Ivesia webberi	10	9,0	7,6	0,1	0,1	4,9	4,4	−0,1	−2,8
Potentilla biennis	333	12,1	5,8	3,8	0,0	8,4	0,5	6,8	−12,1

Temperatures are given in °C and precipitation in millimeters.

#### Climate variables

The four climate variables selected to build the models by analyzing correlation and AUC values were *Standard deviation of mean temperature, Mean temperature in coolest month, Mean daily precipitation in coolest month* and *Mean daily precipitation in warmest month*. Table 4 shows the maximum and minimum values for the four climate variables for all included species. A visualisation of one character mapped onto the final phylogeny is shown in supplementary figure S2.

#### Bioclimatic models, extant species

The projected areas range from the restricted species such as *I. utahensis*, which is an endemic to northern Utah, up to the wide-ranging species *I. kingii*, which finds climatically suitable areas in part of the Great Basin. The niche correlation analysis produced D and I correlation coefficients [57] for each pairwise comparison of species. Coefficients range from 0–1 signifying low to high correlation between age to most recent common ancestor and niche overlap. Mean D was 0.34, whilst mean I was 0.50. These values were consistent within clades (clade A: D = 0.31, I = 0.48; clade B: D = 0.35, I = 0.51) and between sister species pairs (D = 0.27, I = 0.46), indicating a weak relationship between niche divergence and phylogenetic divergence that does not vary between the major clades. This is confirmed by the correlation of niche overlap and age to the MRCA, which gives an insignificant correlation not different from zero (Adjusted r-squared −0.01, p = 0.43).

#### Bioclimatic models, ancestral nodes

Figure 3 shows the fully resolved maximum clade credibility sub-tree of the ivesioids from the BEAST analysis. Ten of the branches have a posterior probability greater than 0.95 and are subjects for further investigation.

#### Projections into palaeoclimatic scenarios

**Figure 4:**
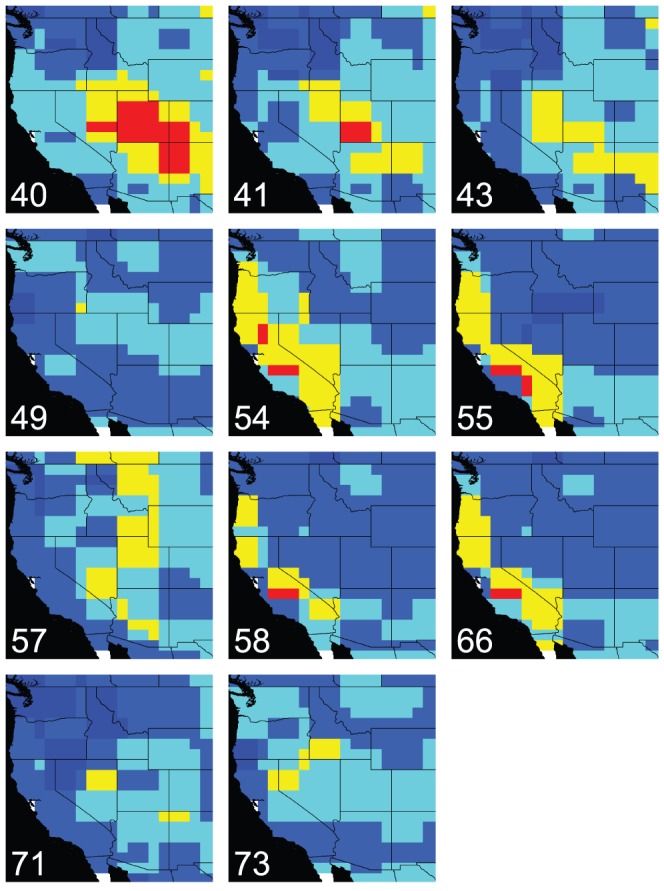
Ancestral bioclimatic models projected into palaeoclimatic scenarios. Red areas are inferred to have had a suitable climate for the ancestral population by four climate variables and areas in yellow by three. Numbers corresponds to the nodes in the ivesioid clade in figure 3.

**Figure 5:**
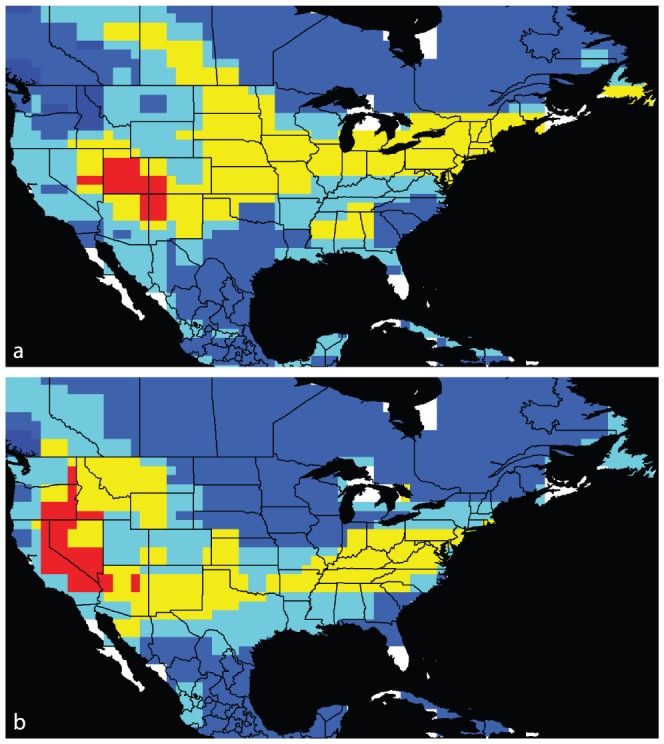
Projections of the ancestral model for the MRCA of the ivesioids and *Potentilla* sect. *Rivales* in (a) palaeoclimate scenario and (b) present-day scenario. Areas in red are inferred by all four climate variables and areas in yellow by three of them.

The reconstructed ancestral climate models, projected into their respective climate scenarios, are shown in figure 4. Node 40 is the MRCA of the ivesioid species and its sister clade *Potentilla* sect. *Rivales*, and hence represents the age of the ivesioid stem lineage. This lineage emerged at 23.4 Ma and is shown to diverge at 17.7 Ma (crown age; node 41 in figure 3). The bioclimatic model for node 40 projected into a climate scenario from 10 Ma indicates an area of suitable climate from where the clade could have evolved (areas marked in red in Figure 5a). Most of Utah, parts of Nevada, Arizona, Colorado and New Mexico are inferred to have had a suitable climate by all four variables.

In node 41, the suitable area inferred by all four climate variables has decreased, but still includes parts of Utah. Due to low support for the topology of the tree, the two well-supported clades (A and B in Figure 3) as well as four taxa with uncertain position (*I. lycopodioides, I. longibracteata, I. jaegeri and I. bailey*) are treated as being derived from this node. Three variables inferred the radiation in clade A (node 71) to Northeastern Nevada, Northern Arizona and New Mexico. The other node with support in clade A (node 73) inferred the Northern parts of the Sierra Nevada, Northwest Nevada and Southwestern Idaho as having had a suitable climate.

The inferred suitable area for node 43, MRCA of clade B, resembles that of node 41, but is weaker (only yellow areas in figure 4, map 43) and slightly more southern. A westward movement of suitable climate is seen in nodes 54, 55, 58 and 66, which have models predicting large parts of the Sierra Nevada and the coast of Northern California. The projected models for two nodes do not corroborate this westward movement of a suitable climate. They are Node 57, with a large part of the Great Basin, Western Montana and parts of Arizona and Canada inferred, and node 49 with only a small part of Southeastern Oregon inferred by three climate variables. Most models also show a weak support for a suitable climate on the East coast of North America and Europe (data not shown).

#### Projections of ancestral models into present-day climate

**Figure 6:**
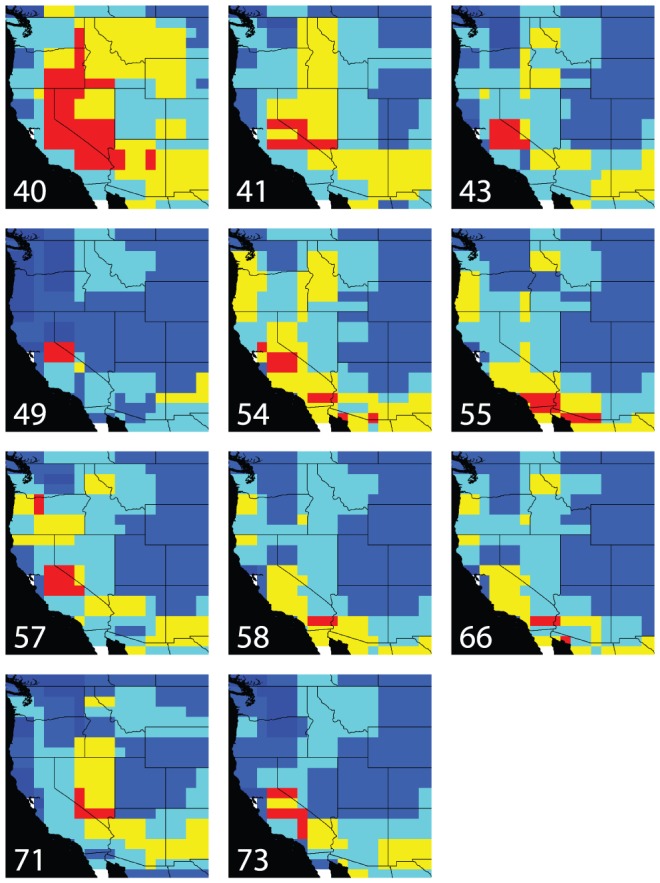
Ancestral bioclimatic models projected into present-day scenario. The maps illustrate the inferred climate preferences of ancestral populations, by showing where this climate type is found today. Areas in red have been inferred by four climate variables and areas in yellow by three. Numbers corresponds to the nodes in the ivesioid clade in figure 3.

Projections of the ancestral model for the MRCA with *P. biennis* (node 40) into present-day climate shows that the ivesioids originate from a climate corresponding to what is now found in the Sierra Nevada, Nevada, Southwestern Oregon and Northeast Arizona (Figure 5b). The preferences for present-day central Sierra Nevada climate prevails for all nodes in clade B (Figure 6; Maps 49, 54, 55, 57, 58 and 66) and are only slightly weakened for nodes 55, 58 and 66. The three latter nodes have an affinity for a climate found around the San Bernardino mountains in the south. As in the projections into palaeoclimate scenarios, there is a shift in climate preferences that includes the type of climate now found along the coast of California, sometime after 12.2 Ma (node 43).

## Discussion

### Phylogenetic inference and Molecular dating

The dated phylogeny of *Rosaceae* is congruent with previous analysis of the relationships in the family (Figures 1 and S1). The topology of the *Potentilla* clade was also congruent with that reported by Dobes and Pauli [9] and Töpel et al. [8] with few exceptions.

### Locality data

Locality data can be highly influential on the model predictions for extant species [58]. It is important to use locality data from all climate regions occupied by the species to be able to create a model that predicts the true climate preferences for the species. Still, less than ten locality points were used in the analyses for *I. longibracteata* and *I. cryptocaulis*. Instead of following the procedure of Evans et al. [7] and manually add extra points from these areas we used only the observed locations, and thereby violated the rule of thumb of only including taxa with more than 10 or 20 data points in the analysis (10 points [19], 10–20 points [58]). The two species are both narrow endemics, with the former known only from Castle Crags (41.17°N, 122.33°W) in the Trinity Mountains, California, and the latter from the summit of Mt. Charleston (36.3°N, 115.6°W) in Spring Mountains, Nevada (Barbara Ertter, personal communication). We manually analyzed the climate data from these areas, and found that adding more occurrence points from the known area of distribution would sample the same values as were already in the model. In effect, each of the two species occupies only one climatic niche (at the scale of our data) in its known area of distribution. The Envelope Score algorithm, used to build the bioclimatic models, only uses the observed minimum and maximum value for each environmental variable to define the bioclimatic envelope of a species. Adding more points from these areas would therefore not change the models. The bioclimatic models for *I. longibracteata* and *I. cryptocaulis* might therefore predict a too narrow area of suitable climate if the distribution of these two species is not limited by the climate. Our primary goal is not to model extant species, but rather reconstruct ancestral models. We therefore believe that it is better to include these minimal models than to exclude these species from the analysis.

### Origin of the ivesioid clade

The crown age of the ivesioid clade (24.3-12.1 Ma; median 17.7 Ma) corresponds to the time when summer drought started to appear in western North America [2]. This supports the hypothesis that the group evolved in response to the Miocene aridification of western North America. Furthermore, the area where the ivesioids are inferred to have originated includes the eastern parts of the Great Basin and the western side of the Rocky Mountains (Figure 5a). This region represents the eastern extension of the present day distribution of the group. *Potentilla biennis*, sister species of the ivesioids, has a distribution from the Sierra Nevada in the west to North Dakota in the east, and from southern British Columbia and Oregon in the north to Arizona in the south. Hence, both species in the ivesioid clade and in the sister group *Potentilla sect. Rivales* can still be found in their optimized ancestral area. In addition, an area outside of the present area of distribution, corresponding to the southeastern parts of North America, is inferred by three variables to have had a suitable climate (yellow area in figure 5a). The hypothesis that the ancestor of the ivesioid clade evolved on the east side of the Rocky Mountains and migrated to the Great Basin, the Sierra Nevada and the coast of California is less parsimonious than an origin and diversification in the Great Basin. The stronger prediction of the Great Basin (red areas in figure 5a) also supports this notion.

The ivesioid clade is inferred to have originated in a climate resembling that of present-day western Nevada, the Sierra Nevada and southeast Oregon (Figure 5b), which indicates that the ancestor had fairly wide climate preferences. However, this result may be due to limitations in the method used for the ancestral state reconstruction and may be unduly influenced by the outgroup, *Potentilla biennis*, which has a relatively wide niche. This is a generic problem with ancestral state reconstruction, but any artificial widening of the ancestral niche preferences would still encompass the ‘true’ niche.

### Diversification in the ivesioid clade

The ancestral niche models for nodes older than 10 million years (node 71 in clade A and node 43 in clade B, as well as the nodes 40 and 41, figure 4), more or less uniformly infer the central Great Basin as the ancestral area. These models are all projected into the same climate scenario, permitting a direct comparison without additional uncertainty caused by potentially conflicting palaeo-climate layers. Furthermore, the geographic areas identified when these models are projected into the present-day climate scenario are also very similar (Figure 6). Hence, the result from our analyses suggests that the diversification of the group and the emergence of the two clades A and B at approximately 17.7 Ma was not driven by climate change or a shift in climate preferences. This is supported by the low correlation between age to MRCA and niche overlap, and uniformly low niche correlation within and between clades. The split may instead have been associated with a shift in pollination syndrome.

Clade A consists of species with flowers that have shallow hypanthia and narrow filaments. Their morphology points towards a pollination syndrome involving small flies and beetles [59].

In contrast, clade B mostly consists of species with wide and flattened filaments, forming a cone on top of a deep hypanthium and are pollinated by bees or bumblebees [14]. Most species in genus *Potentilla* have shallow hypanthia and narrow filaments, and an adaptation to a bee pollination syndrome in clade B could have been an important force for this split.

#### Clade A

Only one clade with a posterior probability greater than 0.95 was found in clade A. It includes the two species *Ivesia santolinoides* and *I. unguiculata*, which do not occur in the Great Basin. Instead, these species have the most westerly distribution of species in clade A, and are only found in the Sierra Nevada and adjacent mountain ranges. The rest of the species in clade A are mainly confined to the interior of the Great Basin. From the MRCA of clade A and B (node 41), and further into clade A there is a narrowing of climate preferences, and a more westerly area of suitable climate inferred between 10.7 Ma (node 71) and 2.0 Ma (node 73). Projecting these models into present-day climate shows that the optimized climate models only change slightly (Figure 6), and the detected westward shift of suitable area is probably due to differences in the underlying paleoclimate scenarios used for the different nodes, thus not representing a change in climate preferences.

#### Clade B

A similar pattern is seen in clade B. The ancestral area with a suitable climate is inferred to be the interior of the Great Basin until 7.6 Ma (Figure 4; maps 41, 43 and 57) for at least part of the clade. Furthermore, projections into present-day climate demonstrate that climate preferences of the ancestral nodes remained relatively stable until that time (Figure 6; Map 41, 43 and 57). At 4.5 Ma we find the earliest indication of preference for the climate of coastal California (Figure 4; Map 66). This type of climate preferences appears in several places in the tree after 4.5 Ma (Figure 4; Maps 54, 55 and 58). Hence, a westward migration, as seen in clade A, is also inferred to have happened in clade B, but continued past the Sierra Nevada to the coastal areas of California. The Mediterranean type of climate of this area emerged approximately 10 Ma [2]. It is therefore reasonable to believe that species in clade B have found suitable habitats in the coastal regions of California on at least two occasions between 12.3 Ma (node 43) and 4.5 Ma (node 66), and between 7.6 Ma (node 57) and 1.7 Ma (node 58).

#### Niche conservatism

We observe a general pattern of niche conservatism amongst earlier lineages, until around 7,5-5 Ma (e.g node 57 in figure 3). There follows a greater amount of niche partitioning amongst related lineages, including a transition towards the coastal Mediterranean type climate in parts of clade B. This partitioning is evident for extant taxa as there are low levels of niche overlap between sister species. If sister species shared more similar niches we would expect to see a pattern of correlation between niche overlap and age to MRCA (i.e. that more closely related species have more similar niches), but this is not the case. The mean niche similarity within clades A and B is similar to the overall niche similarity for all species, so there is no major niche differentiation between clades. Many species pairs in clade B, such as *I. shockleyi*+*I. sericoleuca* and *I. kingii+I. cryptocaulis*, follow a schizo-endemic distribution pattern, i.e. one wider ranging species sister to a narrow endemic, but these sister groupings receive low support. This pattern has been reported for a number of plant groups in the Mediterranean region [60], and has been interpreted as the wider-ranging species being progenitor to the local endemic. Our results corroborate the generality of this pattern, that should be especially important in areas containing distinct micro-habitats (e.g., moist rock crevices in the middle of a wide arid zone, as observed for the ivesioids). We suggest that this may be an underestimated process in plant evolution, which could potentially explain at least some of the plant species richness observed today as well as the uneven distribution of certain species as compared to others that are closely related.

## Conclusions

The phyloclimatic evolution of the ivesioids, inferred here, provides temporal and spatial support for the hypothesis that this group evolved in response to the late Tertiary development of dry conditions in western North America. The age of the MRCA of the clade (24.3-12.1 Ma; median 17.7 Ma) at Early-Middle Miocene coincides with the time when summer drought began in western North America. The hypothesis is further supported by the fact that the eastern parts of the Great Basin and the western slopes of the Rocky Mountains are inferred to have been the ancestral area of the clade. No other part of North America is strongly inferred to have had a suitable climate for the ancestor of this node; thus, migration into the Great Basin from areas not presently occupied by ivesioid species is unlikely.

A shift in pollination syndrome possibly led to diversification of the ivesioids at approximately 17.7 Ma. The resulting two clades experienced a westward range expansion from the foothills of the Rocky Mountains and the central Great Basin to the Sierra Nevada between 10.7-2.0 Ma, in clade A, and on at least two occasions between 12.3-4.5 Ma and 7.6-1.7 Ma in clade B. After a Mediterranean type of climate became established on the coast of California ∼10 Ma, several lineages crossed the Sierra Nevada and found new suitable habitats to exploit. Our results thus suggest that the evolution and current distribution of this morphologically aberrant and diverse group to a large extent has been influenced by past climate change.

## Supporting Information

Figure S1 **Same molecular chronogram of Rosaceae as shown in** **figure 1** **, but also including species names.** Maximum clade credibility tree obtained from 25000 post burn-in Bayesian chronograms generated in BEAST, with median branch lengths. Grey bars at nodes represent 95% Highest Posterior Densities of node ages. The red dots indicates age constraints used for the analysis; (1) The split between Rosales and Fabales was constrained to an age of 104–115 Ma based on a previous analysis [31], and (2) a *Crataegites borealis* fossil was used to set a conservative minimum age of 85.8 Ma on Rosaceae [32], [34]. Subclades of Rosaceae were calibrated using fossil data from (3) *Neviusia*, 48.7 Ma [35], (4) *Chamaebatiaria*, 26.85 Ma [40], (5) *Holodiscus*, 34.1 Ma [37], (6) *Spiraea*, 48 Ma [35], (7) *Rosa*, 34.1 Ma [38], (8) *Fragaria*, 2.5 Ma [45], (9) *Potentilla* 11.6 Ma [42]. A uniform prior with a maximum age of 115 Ma was used for all calibration points. Also indicated are the tribes of Rosaceae (species highlighted in blue and yellow) as well as the ivesioid clade highlighted in red. Time scale from [61].
(EPS)

Figure S2 **Phylogenetic tree of the ivesioid clade with all node numbers indicated.** Nodes and tips are coloured according to the character state of precipitation in the coldest month, with lightest shades indicting the lowest precipitation values.
(EPS)
